# Dr. Yeats on Ischuria

**Published:** 1813-05

**Authors:** G. D. Yeats

**Affiliations:** Bedford


THE
### Medical and Physical Journal.

5 OF VOL. XXIX.] MAY, 1813. [NO. 171.

*To the Editors of the Medical and Physical Journal.*

GENTLEMEN,

HAVING been lately engaged in looking over the pages of some of our older writers on the subject of Ischuria, I have found repeated mention made of vomiting of urine: I shall, therefore, be obliged to you to insert in your Journal the following remarks on Ischuria, in which the various ways by which the constitution relieves itself, when the flow of urine is obstructed, are adverted to and considered. I wish more particularly to draw the attention of your readers to the extraordinary symptom of urinous vomiting; and to show, that, although rare, it has on many occasions undoubtedly occurred, and also that it admits of easy explanation from the laws of the animal economy. I beg the remarks may be considered as merely cursory, and as the embryo of what may, probably, hereafter be the subject of a more enlarged publication.

Nosological writers designate a suppression or retention of urine by the name of ischuria, the etymology of which, from Greek derivation, is easily understood. To this generic term has been added four specific appellations, according to the part where the obstruction is fixed; we, therefore, have ischuria renalis, ureterica, vesicalis, and urethralis. So numerous are the diseases to which the urinary organs are liable, that the accurate and laborious Sauvages has subdivided these four species into forty-four varieties, according to the different causes, and has confirmed the propriety of this subdivision from well authenticated histories.

It is clear, that in three of these species there must be a secretion of urine, but it does not so evidently appear that that secretion must take place to constitute ischuria renalis. Thus then we have two distinct diseases, one where no urine is secreted at all; the other where, after being secreted, its exit from the body is impeded either in the ureters, bladder, or urethra. It is evident, that in the ischuria ureterica, the obstruction may exist in any portion of the ureters, either at their termination in the bladder, or at their commencement in the kidneys: in the latter case, the disease is likely to be confounded with the ischuria renalis, in which, to constitute the genuine disease, there must be a total cessation of the secretion.

secretion. Tulpius relates a case of ischuria in an English clergyman, on opening whose body after death, the pelvis of the left kidney was found considerably enlarged, and filled with urine, and the bladder perfectly empty. The obstruction here was at the origin of the ureter, where it begins to be narrowed from the pelvis. In another case of a person who died of a stone in the bladder, one kidney was found destroyed, and the other so greatly distended with urine, that it seemed to supply the place of the bladder, which was completely empty; ut videretur supplere vicem vesicæ, quam etiam plane vacuam et omni liquore destitutam, at ureteres tam amplos ut in se haud gravatè admisissent digitum humanum.*

Here the impediment to the free passage of the urine existed at the termination of the ureters, which accounts for the distension of these canals: indeed the accumulation of urine in the ureters has sometimes gone on to such an extent as to have increased the size of these ducts to a very considerable magnitude. "Aperto cadavere," says Sauvages of a girl who died of ischuria, "inventi sunt ureteres ita dilatati, ut colon adulti crassitie æquarunt."†

Obstructing causes also often exist in the neck of the bladder, and further along the urethra, producing ischuria vesicalis and urethralis, instances of which are found in Sauvages, Morgagni, and Baillie. In these cases, for the formation of the disease, there must have been a previous secretion of urine; but I consider the ischuria renalis to be a disease arising from a total suspension or annihilation of this secretion. In all cases of this kind, death very soon takes place, except that secretion be speedily restored. "Periculosissima est ei morbi species," says the venerable Heberden, "in qua renes ipsi prorsus otiantur, et nullum humorem ex sanguine eliciunt."‡

In the cases of ischuria where urine has been found in other cavities than those of its proper organs, some professional men of eminence have given it as their opinion that the vicarious organ has secreted the fluid. Dr. Senter has so expressed his belief in his account of a very singular case.§

* Nicolai Tulpii Observ. Med. pp. 164-190.

† Sauvages Nosologia Method, vol. ii. p. 527. See also Baillie's Morbid Anatomy, art. Calculi of the kidnies.

‡ Commentarii, p. 238.

§ An Account of a singular Case of Ischuria in a young Woman, which continued for more than three years, during which she frequently voided it by vomiting. Transact. of the Coll. of Philadelphia, p. 96. This curious case is well worthy an attentive perusal.

Dr.

Dr. Cheyne more than hints at it. Dr. Home delivers it as his doctrine, and Morgagni is of the same opinion. My conviction to the contrary obliges me to dissent from such respectable authorities. I do not think it at all probable that either the stomach, the brain, or the salivary glands, can secrete urine. Each organ is constructed for its particular secretion, and if any other secretion than its own is found in any of them, it is from a totally different cause. Nondum prolata sunt exempla humoris cujusvis secreti, in organo non suo formati; nam alia prorsus ratio est humoris jam formati, inque massam sanguinis denuo resumpti, et tandem ad aliena organa cum sanguine delati.* The liver does not secrete saliva, nor the salivary glands bile: in those cases then in which urine has been found in other cavities than those of the urinary, the kidnies have continued to secrete that fluid. Since then these other organs do not secrete the urine, it becomes a question, in what way does the urine get there? The explanation of this circumstance will arise from considering what are the effects produced, when, after the urine is secreted, it is prevented from being discharged out of the body. Very analogous effects will be the consequence, I mean constitutionally, whether the urine be obstructed in the ureters only, or in the bladder also. I am looking to the general effects. As this will lead me to the discussion of urinous vomiting, I am desirous of premising concisely the laws of the lymphatic circulation, as I believe it is to be explained only upon that system.

It is now above two hundred years since a new system of vessels, distinct from arteries, veins, and nerves, was discovered;† but it was not till a considerable time after this period, that the nature of their structure and functions was rightly understood. These were gradually developed by the arduous labors of Bartholin, Rudbeck, Jolyffe, and many others, down to Hewson and Cruikshank. It was supposed that there were two distinct sets of vessels, one arising from the intestines, and carrying only chyle to the blood; the other from various parts of the body, and transmitting only lymph:‡ the former were called lacteals, and the latter lymphatics. Their functions and structure, however, are now known to be similar, and the whole set of vessels is now denominated the absorbent system. The ap-

* Conspect. Med. vol. i. p. 377.

† By Asellius in 1622.

‡ See this subject treated at large in Observations on Modern Discoveries, by the Author. Sold by Callow.

pellation of lacteal is, however, retained, to distinguish those vessels which transmit chyle. We all know that the different cavities of the human body are furnished with a set of vessels termed exhalents, which are constantly pouring into them various fluids, chiefly for the purposes of lubrication. Hi igitur humores videntur per minutas arterias, vel in cava corporis, vel in pulmonem, vel in cutem hiantes, a sanguine decedere, et fortasse mutari, vel omnino formari, vi nondum satis explorata.* It is also a fact, established from decided experience, and upon the best authorities, that all the cavities of the body are supplied with absorbents, which carry off the fluids effused into them by the exhalents; and there is no fluid secreted or poured out into these cavities, which is not on some occasions absorbed and carried back into the general circulation. Were this not the case, the constant effusion that is going on into shut cavities, would soon accumulate to a dangerous and fatal extent. There is then a constant round observed of exhalation and absorption.† These exhalents also, from different causes, often pour out more than their usual quantities of fluids in a given time: the consequences are, if this effusion takes place into shut cavities, dropsical swellings; if into cavities, or on surfaces possessing excretory orifices, diarrhœa, profuse discharge from the urinary organs, aqueous vomitings, eructations of much fluid, &c. &c. supervene. Those cavities likewise which possess orifices for excretion are also numerously supplied with absorbing vessels, as the urinary and biliary bladders, the uterus, and kidnies; and, notwithstanding they have outlets of their own for carrying off their fluids, these fluids are nevertheless at times taken up from their cavities by the absorbents, undoubtedly for the purpose of obviating the distress and fatal consequences of accumulation, should there be any impediment to the usual and ordinary discharge. But it is in morbid collection of fluids, and in morbid growth of the solid and softer parts of the body, that the extraordinary powers of the absorbents are frequently seen. It is thus that aqueous and purulent swellings, venereal nodes, and enlarged glands, disappear. It is thus that the body becomes discolored in jaundice, and the urine absorbed in ischuria. From the same cause it is that mercurial preparations taken into the stomach, whiten gold in the pocket; and that sulphur consigned to the same viscus, will pass

* Conspect. Med. vol. i. p. 381.

† See an excellent account of the lymphatic circulation, in Mr. Cruikshank's valuable work on this subject, p. 93.

through through the skin. The origin then of the absorbents is very clear, and the necessity for their existence, and for that origin, requires no demonstration. Without them we should become a lifeless mass—a mass of stagnating corruption.

It becomes a natural question, that, since the absorbents are so constantly and actively employed in carrying off fluids which have served the purposes of the economy, and in removing morbid growths and accumulations, what becomes of these matters, and in what way do the absorbents get rid of them? Our knowledge of anatomy teaches us that all the absorbents converge to one part, the thoracic duct,* through which their contents are conveyed into the general circulation: if excrementitious matter, to be ultimately thrown out by the different emunctories; if chyle, to be applied to the nutriment of the body. Such are, concisely, the general laws by which the absorbent system is regulated. The only excrementitious discharges of the human body are the urine, the sweat, the fæces, and the halitus pulmonum. It is evident that these discharges are thrown off as useless, and, if retained, would prove noxious to the constitution. The extraordinary balance that takes place between these discharges, is a curious but well attested physiological fact.

It is daily seen in the diarrhœa, and increased secretion of urine which succeed to suppressed perspiration, and we also know that profuse sweats diminish the secretion of urine, and render the bowels costive. Mirum inter varias secretiones observantur æquilibrium, ita ut pari fere ratione ac aliæ augentur, aliæ minuantur, quo cautum est, ne corpus adeo facile et subito ut aliter fieret exhauriatur. Hoc imprimis notabile est inter halitum cutis et sudorem, et urinam, et excretionem alvi, quamvis in aliis secretionibus idem sæpe observetur.† Clinical observations supply us also with the fact, that the discharges by the intestines, the skin, and the urinary organs, are vehicles of matters which pass through the body unchanged, and therefore they partake not in the least of the nature of the discharges; consequently the organs which secrete or digest these discharges, produce no effect whatever on these matters; they are merely past on as useless or noxious with other excrementitious contents. Thus we often see undigested substances come away with the fæces, sulphur, &c. transpire through the skin with the perspiration, and nitrous salts, the coloring matter of rhu-

* Some of the lymphatics terminate at times directly in the subclavian and jugular vein, without going to the thoracic duct.

† Conspect. Med. vol. i. p. 389.

barb, barb, &c. pass off with the urine. There is no doubt, then, that these three excrementitious discharges are frequently the vehicles of whatever the absorbents may take up from all parts of the body, as noxious or useless to the constitution; and, as far as our knowledge of anatomy goes, these matters can arrive at the different outlets in no other way than through the medium of the circulation, except what passes off immediately from the stomach through the intestinal canal. These doctrines being premised, doctrines which have received confirmation from accurate clinical experience, it is now to be considered what effects are produced on the constitution by an accumulation of urine in the ureters and bladder, when there exists an obstruction to its discharge out of the body by the natural passage. The first sensation would undoubtedly be pain from over distension. According to a law of the lymphatic circulation, the absorbents would set to work to relieve the constitution, the urine would be absorbed and carried into the general circulation, to be ultimately thrown off by the exhalents on some other parts, where in a great many instances relief is obtained and life saved, in others death is the consequence. The result will vary according as the urine is thrown off by the skin, by the intestines, by the stomach, by the navel, by the mouth, or deposited in the cellular membrane, and in the ventricles of the brain. Why the exhalents should pour out in these different ways the accumulations they may receive from the absorbents, I do not pretend to know, except it arises from the circumstance of a greater quantity of the circulation being determined to a particular part from some previous cause of excitement; neither will I take upon me to determine why the absorbents do not at all times remove the urine from an over-distended bladder. The same thing often happens in other cavities of the body; an accumulation of fluids is not always removed by the absorbents. "There is another fluid," says Mr. Cruikshank, "which the lymphatics also take up on particular occasions, and carry into the blood; I mean the urine. I am perfectly confident of this from attending to what has happened to myself. I have had the strongest calls to make water, and felt that the bladder was full; but not having it in my power to quit the company, the symptoms in some little time after have gone off. In an hour or two after, on attempting to make water, I found that the bladder contained little or none. I have not a doubt of urine being absorbed and carried into the blood-vessels."* The explanation, then,

* On the Absorbent System, p. 111.

of of the manner in which urine finds its way to other cavities and outlets than those to which it naturally belongs, is not, I presume, attended with difficulty. When an obstruction to its usual discharge occurs to any extent, the absorbents carry off the urine into the general circulation, and it is poured out by the exhalents in the various ways already stated; if into the stomach, the urinous vomiting takes place. But, however disposed the reader may be to consider this explanation as incorrect or true, the following well-authenticated facts of the urine, when suppressed, being thrown out in the ways above-mentioned, merit attention; and my professional readers are at liberty to form what theory they please, according to the physiological ideas they may have imbibed. The facts are indisputable; they are stubborn things, against which no theory can prevail.

The different ways in which the circulation has got rid of urine carried into it by the absorbents, is a matter of curious research, and show the extraordinary preserving powers with which our constitutions are gifted under inconvenience and distress; constituting the vis medicatrix naturæ of the schools of physic: undoubtedly this vicarious discharge of urine is to be considered in this light; and I see no difficulty in understanding how the urine should be discharged in this way, any more than any other absorbed fluid which has become useless or noxious to the constitution. The reader will easily call to his recollection instances of dropsies, which had resisted powerful remedies, being speedily cured by a spontaneous vomiting of very large quantities of fluid. A spontaneous diarrhœa, and copious sweats, have done the same thing. Why the fluids thus suddenly and rapidly absorbed into the general circulation should take these modes of exit, is not satisfactorily accounted for; except that the urinary organs are too torpid from previous disease to carry off such a sudden efflux of fluids; and why the absorbents, after having been long inactive, should suddenly assume an energetic action, is a question which may still furnish a subject for scholastic disputation; but I should think that nobody could deny that the fluids which the exhalents had in such instances poured out into the stomach, intestines, and on the skin, are the same which constituted the dropsical swellings thus suddenly disappearing.

In the fifth volume of Med. Comment. p. 437, Dr. Johnston, of Kidderminster, relates the case of a gentleman under suppression of urine, whose skin was covered for several days with ammonia muriata, a salt with which the urine is impregnated. The fluid parts, being evaporated with he perspiration, left, no doubt, a saline deposition on the skin,

 similar similar to what we daily see in our chambers. In Morgagni will be found also cases of urinous sweats, so strong and fœtid, that the smell could scarcely be endured. A young woman, in whom the urine had been long suppressed, was given up as lost; when a sweat, having a strong urinous odour, burst forth in great quantities. During the continuance of these urinous sweats, the patient was much better; but when they ceased, she died in a few days of hydrothorax. The dissection of this case is not given; but I have no doubt that had an examination taken place, an urinous effusion would have been found within the chest. There are many cases of a cutaneous discharge of urine mentioned by Bartholin, Malpighi, and Vallisneri.* The breasts of women have been occasionally outlets for the discharge of urine. In the Journal de Medecine (I relate this upon the authority of Haller) is given a case of this kind: per mammas lotium virgo reddidit, quæ absque naturæ alvique ostio nata; and he adds what is very remarkable, and shows how long life will exist under a vicarious discharge, pubertatis tamen tempus adtigerit.† Platerus relates a case, as recorded by Sauvages, of a girl thirteen years of age, who being seized with a suppression of urine, there supervened a very copious serous discharge for several days from the right ear, without suffering any inconvenience: interim bene habitat puella neque de ullo affectu conquerebatur. Upon the return of the urinary discharge, the serous defluxion from the ear ceased, but returned upon the urinary discharge being again suspended. The urine was at last permanently restored, and the girl did well: nihil exinde mali perpetiente puella.‡ Vandermonde gives the history of a girl who, after having for eight months labored under exquisite hysteric convulsions, was seized with a stoppage of both the urinary and alvine discharges, which resisted every remedy for three months. The girl recovered, but, during the suppression, deficientum lotii excretionem supplebat perspiratio cutanea.

Upon the authority of the same physiologist, whom Sauvages calls *laudatus auctor*, I collect the curious history of a

---

* Morgagni de Urinæ Suppressione.

† Haller, p. 371.

‡ P. 526.—"The extraordinary discharges which sometimes take place from other secretory surfaces, ought not to be overlooked. A most remarkable case lately occurred in Dublin, in which a fluid was discharged from the meatus auditorius externus in an enormous quantity—I am afraid to say how much. This fluid contained muriat and phosphat of soda, and plenty of albumen, and it precisely resembled serum."—*Private letter to the Writer.*

woman woman of fifty, who labored seven years under a total stoppage both of the urine and fæces: interim excretionum harumce defectum supplebant sudores universales copiosissimi fœtidissimi nunc die quolibet, nunc quovis biduo triduove erratice recurrentes et ad duas tresve horas continuati. At the end of this period the fæces and urine began to be discharged spontaneously, although she had frequently used the most powerful medicines in vain; and she lived six or seven years after in health.*

The intestinal canal, a portion of which is the great reservoir of excrementitious matters, forms also a vent from which urine is discharged when the natural outlet to that secretion has been obstructed; and this takes place when there has been no opening formed by ulceration between the rectum and the bladder. Puer duodecim annorum, says Baglivi; postquam per septem dies urinæ suppressione laborasset, eandem demum excrevit per alvum et sanavit.†

"Early in the month of January, 1787," says Dr. Senter, in the history of his singular case, mentioned more particularly hereafter, "from some cause unknown, she could not be relieved by the instrument, nor could she vomit up her urine for several days, when it past off by the navel for three days successively. Early in the spring, 1789, her urine began to pass per anum, loaded with the same kind of gravel that had come away by the catheter."‡ Groenvelt, who, I believe, was prosecuted for prescribing cantharides internally, also relates instances of the discharge of urine per anum. Cum duodecimo die, says Haller, quoting Groenvelt, suppressæ urinæ jam gravis lethargus ingenerit, urina multa per alvum cum levamine egesta est; and, undoubtedly, if this state of stupor had not been removed by the discharge of urine per anum, the exhalents would have poured it out on the brain, to relieve the turgid vessels within the cranium, and the patient would have died apoplectic. Cases of a similar kind are given by Pechlin and Rhodius.§ Vidi senem quendam, says the celebrated Hildanus, in a letter to Horstius, urinam omnem per podicem sine insigni molestia excernentem, in quo post mortem, ductum urinarium circa sphyncterem scirrho occlusum esse reperi. The same surgeon mentions also the fact of a young woman of twenty, who past her urine at the umbilicus.‖ Morgagni, after relating

* Sauvages, vol. ii. p. 526.

† Georgii Baglivi Libri Duo de Praxi Medica, p. 123.

‡ Pp. 103—109.

§ Haller, p. 370.

‖ Gregorii Horsti Observ. Medicinal. p. 516.

several cases of the discharge of urine per anum, where no passage could be discovered between the rectum and bladder, adds, but lest it should be supposed that some mark of preternatural perforation might have been overlooked in these cases, attend to what the celebrated Reusnerus relates of a case: there was no urine in the bladder which could make its way by force into the intestine, and yet, on the seventh day after the ischuria, "urine, which was similar to what is naturally excreted in color, smell, and quantity, was discharged from the intestines, without any discharge of the intestinal fæces at the same time, without any pain or alteration, and that three or four times for some days together, till all of a sudden it was again discharged by the penis, without the least pain or troublesome symptom." Here not only was the urine thrown off from the intestines, but it came away perfectly free from fæces. Morgagni naturally enough could not easily account for this; he, however, did not dispute the fact; for he immediately, from his own knowledge, relates the case of a priest, endued "with an excellent natural disposition, with a probity worthy of his office, and with manners that were always exemplary," who for a long time past his urine per anum: and "this fluid, which generally flowed from the intestine without any of the excrements, continued to flow from thence even to the day of his death, which was brought on by quite a different cause." Morgagni supposed the discharge of urine to take place in this way, in consequence of the intestinal glands secreting it.*

The next organ into which the urine has been frequently known to be deposited, and in considerable quantities, is the stomach. Cases of this kind are numerous; so much so, that Sauvages has formed a species in his nosology under the name of Vomitus Urinosus. We meet with this in the writings of Vander Wiel, Horstius, Haller, Morgagni, and many others, down to authors of our own days; and the urine has been thrown up from the stomach for a considerable time, sometimes incorporated, and at other times pure and unmixed, with the contents of the stomach. An elderly gentleman labored under a suppression for sixteen days, in consequence of calculi: interea tamen, says Vander Wiel, per illos dies ipse præ nimia oppressione urinæque impedito exitu hanc, et salsissimam, aliquoties vomitu ejecit.† Horstius, to whose work I have already referred, relates a

* I could not procure the Latin edition of Morgagni. Pp. 543—544 of the translation.

† C. Stalpart. Vander Wiel Observ. Rarior. Med. p. 218.

very very singular case, in a letter to Dr. Firkman, physician at Worms:—Pueri quem ante 9 vel 10 circiter annos Darmstadii vidi, qui pene testibusque penitus carebat, cute istius loci totaliter consolidata, quem defectum puer ex morsu porci dicebatur habere, quo primo ætatis anno genetalia penitus ablata fuerunt, ex incuria post, membro isto totaliter consolidato. Hic puer annorum circiter 12 tunc temporis, ubi Darmstadii a me videbatur candide referebat quod quotîdie bis compressionem aliquam circa regionem vesicæ sentiret, subsequenti mox vomitu salsæ aquæ.* About the time of the publication of this work of Horstius (1620), the attention of the profession was a good deal excited in Germany respecting a singular case, which employed the pens and exercised the ingenuity of the first physicians and surgeons of the day. A poor woman fell with a stick of wood in her hand in such a manner, that she inflicted a severe wound upon the passages leading to the uterus and bladder. Either from poverty or modesty, or both, she failed to apply for surgical aid; the consequence was, that a complete union of the parts took place from adhesive inflammation; after which she vomited up and past her urine per anum. Upon this very singular case a long correspondence took place between Drs. Fittich and Firkman, physicians at Worms, Dr. Horstius, of Ulm, and the celebrated Fabricius Hildanus, surgeon at Bern. Great doubts were entertained of the fact; the consequence was, that Horstius minutely examined the girl, with Adam Stoss, a surgeon, to ascertain the truth; and with the intention also, if possible, of dividing the coalesced parts, and renewing the natural passages of the urine. On inquiry the facts were found as stated; and, upon consultation, the operation was thought too hazardous to be attempted. The poor girl was therefore left to her fate. No discovery was made of any passage for the urine from the bladder to the rectum, as Hildanus very judiciously stated might be the case. He did not, however, disbelieve the fact; for, in the letter mentioning this as the probable outlet, he states the case of a girl who, from an obstruction at the neck of the bladder, past her urine at the navel; and of a man who, from the same cause, past it per anum. Four years after the accident, the poor woman was still living, and passing both her urine and menses per anum. It would appear that Horstius had requested Dr. Muller to keep his eye upon the woman, for he thus writes to Horstius: Statum, istius mulieris in pudendis vulneratæ, quem pro tempore, post tot annos scire desideras, talis est, qualem reliquisti. Vivit adhuc sed miserè satis,

* P. 513.

sicut ex ore ipsius proprio intellexi, nec aliter urinam atque menses quam per alvum, sæpius non absque gravissimis doloribus, excernit, muliebribus penitus adhuc consolidatis.* Haller, too, refers to several great authorities for cases of urinous vomiting. One of our own profession was afflicted with this nauseous disease. The celebrated French surgeon Lanfranc suffered under it. Eadem (says Haller) quæ per alvum, urinæ via etiam ad ventriculum ducit. Celebris olim chirurgus Lanfrancus ipse, cum calculo laboraret, lotium vomuit. Laweria illa, olim per suum morbum notissima, aliquoties urinam vomitu reddidit.† He also directs the attention of his readers to similar cases published in the History of the Royal Academy of Sciences at Paris in the year 1715. They are the cases of a nun and a monk. The former, after having been long harassed with hysterical complaints, was seized with a suppression of urine which lasted forty days, during which she vomited that fluid. At the end of this period the urine returned to its natural channel. The urinous vomiting, however, soon recurred, and had continued thirty-two days longer, when the account was drawn up by Dr. Marangoni, physician at Mantua. A very remarkable circumstance in this case is, that the urine was vomited unmixed with the food, although the vomiting sometimes occurred soon after her meals. Lemery, the French chemist, was acquainted with the case of the monk, who is stated to have vomited a urinous fluid. Vallisneri, as Morgagni states, saw a girl who, after the tenth day of suppression, vomited a fluid of the color, taste, and smell, of urine. She lived for some time in this way, till it occurred to Vallisneri to have recourse to mercury to overcome the obstruction, and it had the desired effect. Also, says Morgagni, another physician saw a woman labor under this suppression and vomiting for fifteen months, till the calculus being discharged, the ischuria and vomiting of urine went off.‡ A great number of similar instances might be given were it necessary; and, if the reader will take the trouble of turning over the pages of Morgagni, Haller, and Sauvages, his curiosity will be amply gratified. Similar cases of more recent date are to be found in the annals of medicine. §

---

* P. 517. † P. 371. ‡ P. 453.

§ Dr. Cheyne, who has published so well on several subjects, after having quoted Portal on the subject of urinous deposition on the brain, adds, "And yet this is scarcely more extraordinary than the case of the girl I saw in 1797. She vomited, at stated times, a fluid with all the sensible qualities of urine; the discharge by the urinary passage having long been suspended."—On Apoplexy, p. 207.

A very

A very accurately drawn up case of ischuria, attended with urinous vomiting, will be found in the Transactions of the College of Physicians of Philadelphia, by Dr. Senter. The following is an abstract of the case:

A young woman, after having been in a bad state of health for about a year from sympathetic irritation of the lungs, in consequence of diseased stomach, at times vomiting blood, was seized with a suppression of urine, accompanied by such an irritable state of stomach, that nothing remained upon it but solid opium for ten weeks. On the sixth day of the suppression, she brought up urine from the stomach, with which was subsequently mixed gravelly matter. "I saved," says Dr. Senter, "the water that she brought up this way, and compared it with what I drew off, and found it the same in every respect." The urinous vomiting continued for three years, during which it also at times past per anum, and at others from the navel. The water was frequently drawn off by the catheter, which relieved the soreness felt at the lower part of the belly.* Sometimes, although the catheter remained in the bladder for a whole night, no urine at all would come away, although she was throwing it up in considerable quantities from the stomach.† Many other symptoms of extreme distress harassed this patient. She was at one time paralytic; her frame was shook with convulsions; there was occasionally a vomiting of bloody pus; gravel, as well as urine, was also discharged from the stomach, and, in comparing this gravelly matter with the gravel which came from the bladder by the instrument, "I could observe," says Dr. Senter, "no difference either in the color or consistence of them."‡ It is worthy of remark, that, during the progress of this girl's illness, "she twice passed a small quantity of urine through the urethra, in consequence of being frightened once by thunder, and the second time by the falling of a window in her room."

As far as I collect from reading the case, these were the only times, during the three years that the ischuria prevailed, that she passed urine by the natural passages, except with the assistance of the instrument; and, notwithstanding a

---

* The ischuria vesicalis at this time prevailed.

† The ischuria ureterica seems to have been present at this time. See Morgagni for several cases of enlarged ureters. See also Sauvages and Cullen for the symptomatic discrimination between ischuria vesicalis and ureterica.

‡ Haller mentions a curious fact: "In nares urina se convertit, raro in exemplo, cum in paralysi vesica officio non fungeretur; arenosæ enim glebulæ naribus adhæserunt." P. 371.

considerable considerable quantity of urine was thus repeatedly drawn off, the relief obtained made no permanent impression on the urinous vomiting, as the urine continued to be thrown up from the stomach to the period of her dissolution.

On examination of the body after death, although a great deal of disease was seen, no discovery was made of any channel by which the urine could pass from the bladder to the stomach.

The case is exceedingly curious, and is a complete confirmation of what has been related in medical annals of urinous vomiting.* In addition to the various outlets already stated which the urine has found when supprest, it has been known to be discharged into the mouth and fauces, into the cellular membrane in different parts of the body, and on the brain. Cases of vicarious ptyalism in ischuria are very common in authors. "Unicus ex iis quos vidi," says the experienced Heberden, "urinæ saporem in ore questus est."† In "some cases," says Dr. Home, "it seems to have been secreted by the salivary glands, and then to have retained the natural taste of urine."‡ In a case of suppression of urine mentioned by Mr. Warner, there was so large a quantity of saltish water, tinged with blood, discharged from the mouth, as to threaten suffocation § Salivæ loco, says Haller from Camerarius, de mictu Periodico, fœtidissima urina reddita est, faustu quidem eventu.|| Natali, the preceptor of the celebrated Malpighi, labored under ischuria for many days, during which he perceived the taste and smell of urine in his saliva, and the perspiration partook of the same urinous qualities.¶ Albertini relates the history of a young nobleman, who, laboring under this disease, not only spat up saliva with the taste and smell of urine, but even almost urine itself, as the color added to the taste and smell testified; and the fauces were so overloaded with the afflux of this urinous fluid, that the cheeks and parotid glands were considerably swollen.**

It is very evident that when a great diminution takes place in either the fluid evacuations or secretions for any length of

* The Editors of Medical Facts, anno 1795, have given an account of this case in their 6th vol. (and have subjoined the cases of the nun and the monk from the Parisian Academy of Sciences) but it will be found more fully detailed in the Philadelphia Transactions.

† Commentarii, p. 238; and he very justly adds, Puto renes non cessasse urinam excernere, quamquam exitus illi proclusus erat.

‡ Clin. Experts. p. 295.

§ Cases in Surgery, p. 265.

|| P. 371.

¶ Morgagni, p. 452.

** Ibid. p. 452.

time, an accumulation of fluid must take place in some of the cavities of the body, provided no vicarious discharge is substituted for the supprest one.* Cases of this kind are so common, and the fact so obvious, that it is superfluous to refer to authorities for a confirmation of the truth of the position. It has, however, been seldom suspected that these accumulations, in consequence of suppression, have been formed by the urine itself. The examination of bodies after death has clearly proved this, and it is a fair belief, founded on analogy, that in dropsical swellings, arising from ischuria, the effused fluid partakes of the qualities of urine.

In cases of pure ischuria renalis, where no urine is secreted, it is plain, I think, no urine can be deposited into any of the cavities of the body; but in those cases in which, after the secretion, the suppression occurs, it appears to me very probable, when a considerable accumulation of dropsical swellings takes place, the suppression continuing, that the effused fluids will be found more or less affected by a urinous impregnation. In the majority, if not in the whole, of the cases published by Dr. Home, great hydropic swellings took place: in one case, a spontaneous diarrhœa supervened, and carried off the swellings, notwithstanding the ischuria continued, and ultimately proved fatal. In a letter of Dr. Fittich to Dr. Führman, published in the correspondence of Horstius, already quoted, it is remarked, in a case of suppression of urine of about three weeks continuance, interim toto corpore, maxime vero venis admodum turgidis, quæ ex levi frictione multam aquam resudabant, in quo difficillima et gravis mors ex suffocatione et tumore totius corporis secuta.† Haller too makes observations to the same effect: In cellulosæ telæ cavernulas lotium decessit in exemplo, in quo œdema urinosum ischuriæ supervenit.‡ Sauvages has given the name of anasarca urinosa to these cases, and states, that in one of this kind the swellings were so extensive as to rise to the neck, tumor universalis ad collum usque sensim ascendens, and that the patient recovered, discharging urine copiously, with fainting.§ The supposition that a urinous deposition might take place in these and similar cases, is strengthened by the dissection of a body by Morgagni. A young man died on the 21st day, after having labored under a suppression of urine, and a swelling of the whole body

* See a case in Med. Commun. vol. i. p. 155, in which above one pint and a half of saliva of a saltish taste was spit up every twenty-four hours, from a diminished secretion of urine. The spitting increased in proportion as the urine diminished, and vice versa.

† P. 509. ‡ P. 371. § Vol. ii. p. 474.

during during that time. On examination, the bladder and kidnies were discovered to be sound: the former contained about two pints of urine. In the cavity of the abdomen was found a fluid that smelt like urine, though limpid in appearance. This fluid, being preserved in a glass vessel, separated into many broken parts, like those generally contained in urine.*

The coma which takes place in cases of ischuria, ab urinæ refluxum, as Sauvages calls it, pointed out to professional men, as was imagined, a connection between the renal secretion and the functions of the brain; but the fact is, that this symptom is as uncertain as that the urine should affect any other organ under such circumstances. It depends upon causes which our ignorance of some of the laws of the human economy still keeps in obscurity; but in all great abdominal irritations, the sensorium is always in some degree affected. In the ventricles of the brain of persons who have died of ischuria, urine has been found. "In some rare instances of coma after ischuria," says Dr. Cheyne, "it would appear as if the vessels of the brain assumed an action similar to the healthy action of the kidnies; fluids, with the color and smell of urine, having been observed in the ventricles of the brain;" and gives the following quotation from Portal: J'ai reconnu l'odeur d'urine dans une epanchement d'eau qui s'etoit fait dans les ventricles du cerveau d'un homme mort d'une suppression d'urine.† And Haller says, from the Prælect. Boerhaav., In cerebri ventriculis, post symptomata affecti encephali aqua urinæ fætore, vitiosa, reperta est.‡ Urina repressa, (says my venerable preceptor, Dr. Gregory,) et in sanguinem resumpta, totum corpus inundat et in ipsum aliquando cerebrum effunditur. §

Such are the authorities which confirm by indisputable facts that the urine, when supprest, is not only frequently deposited in different parts of the body, and thrown off at other than the natural outlets, but also that the patients survive and live for many years after such an event; in some the urine has continued to be discharged in such an unusual manner, in others it has returned to its proper channel.

I am, Gentlemen,

Your very obedient Servant,

*Bedford, March* 1813. G. D. YEATS, M.D.

---

* Pp. 451-2. Dr. Lister, in his Exercitat. de Hydrope, mentions a man of fifty, who, being seized with ischuria, an immense swelling of his belly took place. A catheter was introduced, but there was no urine. Diuretics with purgatives cured the man. It appears to have been a case of ischuria ureterica.

† On Apoplexy, p. 207. ‡ P. 371.

§ Conspectus Med. vol. i. p. 377.

